# NIH3T3 Directs Memory-Fated CTL Programming and Represses High Expression of PD-1 on Antitumor CTLs

**DOI:** 10.3389/fimmu.2019.00761

**Published:** 2019-04-11

**Authors:** Yingyu Qin, Yuna Lee, Jaeho Seo, Taehyun Kim, Jung Hoon Shin, Se-Ho Park

**Affiliations:** ^1^Department of Life Sciences and Biotechnology, Korea University, Seoul, South Korea; ^2^ImmunoMax Co., Ltd, Korea University, Seoul, South Korea

**Keywords:** NIH3T3-CM, cytotoxic T lymphocytes, memory precursor, memory CD8^+^ T cells, adoptive cell therapy

## Abstract

Memory CD8^+^ T cells have long been considered a promising population for adoptive cell therapy (ACT) due to their long-term persistence and robust re-stimulatory response. NIH3T3 is an immortalized mouse embryonic fibroblast cell line. We report that NIH3T3-conditioned medium (CM) can augment effector functions of CTLs following antigen priming and confer phenotypic and transcriptional properties of central memory cells. After NIH3T3-CM-educated CTLs were infused into naïve mice, they predominantly developed to central memory cells. A large number of NIH3T3-CM-educated CTLs with high functionality persisted and infiltrated to tumor mass. In addition, NIH3T3-CM inhibited CTLs expression of PD-1 *in vitro* and repressed their high expression of PD-1 in tumor microenvironment after adoptive transfer. Consequently, established tumor models showed that infusion of NIH3T3-CM-educated CTLs dramatically improved CTL mediated-antitumor immunity. Furthermore, NIH3T3-CM also promoted human CD8^+^ T cells differentiation into memory cells. These results suggest that NIH3T3-CM-programmed CTLs are good candidates for adoptive transfer in tumor therapy.

## Introduction

Adoptive cell therapy (ACT) using autologous tumor reactive T cells has emerged as a potentially curative therapy for cancers (1–3). Cytotoxic T lymphocytes (CTLs) are considered as potent lymphocytes that can perform direct lysis of target tumor cells precisely. Nevertheless, a significant limitation for ACT is that repeated stimulation alters functional capabilities of CTLs which can result in defects in survival and function after transfusion (4, 5). Many studies have suggested that memory T cells are superior to effector T cells in antitumor activity due to their long-term persistence and more robust effector functions in response to tumor antigens (6–9). One prominent notion that has been accepted is that once naïve CD8^+^T cells are primed, the majority of effector CTLs will die via differentiation into short-lived effector cells (SLECs) while only a small subset will differentiate into memory precursor effector cells (MPECs) destined to become long-lived memory cells (10–13). In this regard, exploring a proper culture condition to direct the differentiation of tumor-specific CD8^+^ T cells to MPECs may be a promising approach to develop a curative antitumor therapy upon adoptive transfer.

Fibroblasts are heterogeneous tissue connecting cells that play critical roles in wound healing. Fibroblasts are also responsible for the production of extracellular matrix molecules that can act as co-stimuli for T lymphocyte activation (14, 15). Soluble factor(s) secreted by fibroblasts from malignant or non-malignant tissue can enhance T cell IFN-γ and IL17A production (16). Fibroblasts derived factor(s) can also inhibit activation-induced apoptosis of T cells (17, 18). Given these comprehensive effects of fibroblasts on T cells, altering the fate or intrinsic functions of T cells by fibroblasts might have potential to be utilized in an *in vitro* culture system for ACT. Our previous report has shown that soluble factor(s) derived from mouse embryonic fibroblast (MEF) can strongly enhance the effector function of CD8^+^ T cells (19). NIH3T3 is an immortalized embryonic fibroblast cell line. NIH3T3 cells are widely used as feeders to support long-term survival and self-renewal of tissue progenitor cells (20, 21). In this regard, we sought to investigate whether NIH3T3 could affect the function or the fate of CD8^+^ T cells during antigen priming in co-culture conditions. We found that NIH3T3-conditioned medium (NIH3T3-CM) directed CD8^+^ T cells toward differentiation of potent memory-fated effector clones. NIH3T3-CM not only strengthened effector functions of CD8^+^ T cells, but also conferred characteristics of memory cells. Using adoptive transferred model, we experimentally demonstrated that NIH3T3-CM could program CTLs with high capacity in development of long-lived memory cells. In addition, using established tumor model, we found that adoptive transfer of NIH3T3-CM-educated CTLs exhibited dramatical therapeutic effects. This is not only attributed to high persistence and functions of CTLs, but also due to their low expression of PD-1.

## Materials and Methods

### Mice and Cells

Wild type C57BL/6 mice (WT B6, Ly5.2^+/+^) and ovalbumin (OVA)_257−264_-specific TCR (Vα2 and Vβ5) transgenic mice (OT-1) maintained on B6 background were purchased from The Jackson Laboratory (Bar Harbor, ME, USA). Ly5.1^+/−^ (Ly5.1^+^Ly5.2^+^) OT-1 mice were obtained from OT-1 mice that were crossed to congenic Ly5.1^+/+^ B6 mice. Ly5.1^+/−^ OT-1 mice were backcrossed with B6 (Ly5.1^+/+^) to obtain Ly5.1^+/+^OT-1 mice. All mice were 7–9 weeks old at the beginning of each experiment. They were raised in a specific pathogen-free environment at Korea University. Experimental protocols adopted in this study were approved by the Institutional Animal Care and Use Committee of Korea University.

NIH3T3 cells were purchased from ATCC. EG.7 tumor cells expressing chicken OVA were provided by Dr. M. Mescher (University of Minnesota, Minneapolis, MN, USA). Human peripheral blood mononuclear cells (PBMCs) were purchased from ImmunoSpot. T2 cells were obtained from ATCC. NIH3T3 cells were maintained in Dulbecco's modified Eagle's medium (DMEM, Gibco). EG.7 cells, T2 cells, and primary lymphocytes were cultured in Roswell Park Memorial Institute (RPMI)-1640 medium (Gibco). Both culture media were supplemented with 10% heat-inactivated fetal bovine serum (FBS, Gibco), 2 mM L-glutamine, 1% penicillin-streptomycin, 10 μg/mL gentamycin, and 50 μM β-mercaptoethanol (Gibco-BRL). NIH3T3-conditioned medium (CM) was obtained by seeding NIH3T3 cells at density of 1.25 × 10^5^ cells/ml in DMEM supplemented with 10% FBS, 2 mM L-glutamine, 1% penicillin-streptomycin, 10 μg/mL gentamycin, and 50 μM β-mercaptoethanol and cultured for 2–3 days. CM was then collected by centrifuging at 400 g for 5 min followed by filtration through a 0.22 μm pore size filter. It was then stored at −85°C.

### *In vitro* T Cell Activation

CD8^+^ T cells were sorted from OT-1 or WT splenocytes with a MACS column using anti-mCD8α magnetic beads (Miltenyl Biotec). The purity of sorted OT-1 cells was >95%. For K^b^-OVA beads preparation, 1 μg of OVA_257−264_ (Genscript) loaded biotinylated recombinant MHC class I molecules (H2-K^b^), 0.3 μg of biotinylated anti-CD28 antibodies, and 0.05 μg of streptavidin magnetic beads [NEB, S1420S] were incubated at 4°C overnight with rotation. Then 0.5–1 × 10^5^ enriched OT-1 CD8^+^ T cells were stimulated with K^b^-OVA beads in the presence or absence of NIH3T3-CM (v/v, 50%) in 96-well plates at indicated time points for *in-vitro* analysis. For adoptive transfer, 3 × 10^5^ OT-1 CD8^+^ T cells were stimulated with K^b^-OVA beads in the presence or absence of NIH3T3-CM (v/v, 50%) in 48-well plates and 3 × 10^5^ WT CD8^+^ cells were stimulated with plate bounded anti-CD3/CD28 in 48-well plates. After 3 days of culture, cells were harvested and washed twice with PBS for adoptive transfer. For whole splenocyte activation, 2 × 10^5^ splenocytes were stimulated with 100 ng/ml OVA_257−264_ peptides for 2 days in 96-well plates. Goligistop was used for treatment for 4 h before intracellular staining. Human naïve CD8^+^ T cells were sorted from PBMCs using a naïve CD8^+^ T cell isolation kit (Miltenyi Biotec). Then 1 × 10^5^ purified naïve CD8^+^ cells (purity >95%) were activated by plate-coated anti-CD3 (OKT3, 1 μg/ml) and anti-CD28 (CD28.2, 3 μg/ml) in the presence or absence of NIH3T3-CM in the 96-well plates. On day 3, 20 U/ml hIL-2 (animal-free, PeproTech) was added. Purified naïve CD8^+^ cells (HLA-A^*^0201) (2 × 10^5^) were activated by 1 × 10^6^ CMV-pp65 peptide (NLVPMVATV)-loaded autologous PBMC (2000 rad γ irradiation) in 96-well in the presence or absence of NIH3T3-CM. On day 3, 100 U/ml hIL-2 was added. Expanded cells were moved to 48-well plate on day 5. Medium was refreshed every 2 to 3 days. Cells were collected on day 12 and FACS analysis was performed.

### Trans-well Assay

A trans-well insert (Corning) with diameter of 6.5 mm and pore size of 0.4 μm was utilized to physically separate OT-1 CD8^+^ T cells (1 × 10^5^, upper chamber) from NIH3T3 (1 × 10^5^, lower chamber) while soluble factors were allowed to diffuse into the upper chamber. OT-1 CD8^+^ T cells together with NIH3T3 were added to the upper chamber. After 2 days of stimulation with K^b^-OVA beads, IFN-γ and granzyme B producing cells were detected by flow cytometry after intracellular staining.

### *In vitro* Cytotoxicity Assay

Specific killing of target tumor EG.7 was measured using CFSE/7-AAD based flow cytometry assay as described previously (22). Briefly, effector cells (splenic OT-1 CD8^+^ T cells) were activated by K^b^-OVA beads for 3 days in the presence or absence of NIH3T3-CM (50%, v/v) and labeled with CFSE. CFSE labeled effector cells were then incubated with target cells at effector: target (E:T) ratios of 8:1, 4:1, 2:1, 1:1, or 0:1 at 37°C incubator with 5% CO_2_. After 4 h of incubation, cells were washed and stained with 7-AAD to assess dead cells on CFSE negative cells via flow cytometry. The percentage of specific lysis was calculated as follows: %lysis = 100 × (% sample lysis–% basal lysis) /(100–% basal lysis), where basal lysis was lysis of target cells in the absence of effectors. For T2 killing assay, 1 × 10^6^ T2 cells as targets were loaded with 5 μg/ml CMV peptides followed by 30 μM calcein-acetyoxymethyl dye (Invitrogen) treatment. Effector cells obtained from CMV-pp65 specific expansion were incubated with CMV-pp65 peptide-loaded T2 cells at E:T ratio of 10:1, 5:1 or 2.5:1 at 37°C in an incubator with 5% CO_2_ for 4 h._._ The fluorescence of sample supernatant was measured. The percentage of specific lysis was calculated as follows: %lysis = 100 × (sample release-SP) /[MAX + (MM -MT)–SP], where target cells treated with 2% Triton X-100 represented MAX (Maximum release), SP (Spontaneous release) represented target cell alone, MM represented background of medium, and MT represented background of Triton X-100.

### Antibodies and Flow Cytometric Analysis

For flow cytometric analysis, cells were incubated with anti-Fcγ receptor antibody (2.4G2) generated from mice acsite and then labeled with the following antibodies: anti-mouse antibodies TCRβ-Fluorescein isothiocynate (FITC, H57-597), CD25-FITC (7D4), CD69-FITC (H1.2F3), Ly5.2- Phycoerithrin (PE, 30-F11) or FITC (30-F11), TCRVα2-PE (B20.1), CD127-PE (A7R34), CD27-PE (LG.7F9), Tim3-PE (RMT3-23), CD8α-PerCP-Cyanine5.5 (53-6.7), B220-PerCP-Cyanine5.5 (RA3-6B2), KLRG1-PE.Cy7 (2F1), TCRVβ5.1,5.2-Allophycocyanin (APC, MR9-4) or FITC (MR9-4), CD44-APC (1M7) or PE (1M7), CD62L-APC (MEL-14) or APC.Cy7 (MEL-14), streptavidin-PE or APC or APC.Cy7, CD28-biotin (37.51), CD122-biotin (5H4), PD-1-biotin (29F.1A12), Ly5.1-biotin (A20) or FITC (A20), and K^b^-OVA_257−264_-biotin-streptavidin-PE (Lab made); anti-human antibodies CD3-APC (RE613), CD45RA-PE-vio770 (T6D11), CD45RO-FITC (UCHL1), and CCR7-APC efluor780 (3D12).

Intracellular molecule expression was determined following fixation and permeabilization with either Cytofix/Cytoperm (BD Biosciences) or FoxP3/Transcription Factor Staining Buffer Set (eBioscience) with anti-mouse granzyme B-PE (NGZB), Eomes-PE (Dan11mag), Bcl-6-PE (K112-91), IFN-γ-APC (XMG1.2), T-bet-APC (4B10, also react to human), Blimp-1-Alexa Fluor 647 (5E7), or anti-human Eomes PE (WD1982). Flow cytometry was performed using FACSVerse or FACSCalibur device (BD Biosciences). Data were analyzed using FlowJo_V10 (FlowJo LLC).

### Adoptive Transfer Studies

For cell persistence assay, Ly5.1^+/+^ OT-1 CTLs (2.4 × 10^6^) generated in RPMI-1640 medium and Ly5.1^+^Ly5.2^+^ OT-1 CTLs (1.6 × 10^6^) generated in the presence of NIH3T3-CM (50%, v/v) were intravenously (i.v.) co-transferred to Ly5.2^+/+^ B6 WT mice. The persistence of transferred cells in peripheral blood was detected on days 3, 7, 15, and 30. On day 30, frequencies of transferred OT-1 cells that migrated to spleen, inguinal lymph node, bone marrow, and lung were measured.

### Tumor Rejection Assay

To evaluate tumor reactivity of memory CD8^+^ T cells, OT-1 CTLs generated in the presence or absence of NIH3T3-CM (50%, v/v) were i.v. injected to WT B6 mice (1 × 10^6^/mouse). One month later, OVA-expressing EG.7 tumor cells (2 × 10^5^/mouse) were subcutaneously (s.c.) transferred to mice and tumor growth was monitored.

To detect tumor reactivity to established tumors, EG.7 tumor cells (0.5–0.7 × 10^5^/mouse) were s.c. inoculated to WT B6 mice. When tumor grew to 30–50 mm^3^ (usually between 12 to 14 days), CTLs were i.v. transferred to tumor-bearing mice. Tumor sizes were measured every 2 to 4 days. IL-2 co-administration was performed as described previously (23). Briefly, OT-1 CTLs were i.v. transferred to tumor-bearing mice and 2 μg hIL-2 (animal-free, PEPROTECH) was i.p. (intraperitoneal injection) injected to mice once on the day of CTL injection and twice a day on the two following days. Tumor sizes were calculated by determining the length of short (l) and long (L) diameters (tumor volume = l^2^ × L/2). Experimental endpoints were reached when tumor volume exceeded 2,500 mm^3^. For transferred CTLs detection, EG.7 tumor cells (1–1.5 × 10^5^/mouse) were s.c. transferred to WT B6 mice. When tumor grew to 50–100 mm^3^, 1 × 10^6^ *in vitro*-generated Ly5.1^+/−−^ OT-1 CTLs in the presence or absence of NIH3T3-CM (50%, v/v) or combination of Ly5.1^+/+^ OT-1 CTLs (medium alone) and Ly5.1^+^Ly5.2^+^OT-1 CTLs (NIH3T3-CM, 50%, v/v) at a ratio of 1:1 were i.v injected to tumor-bearing mice. At 6 days after T cell transfer, mice were sacrificed and analyzed.

### Statistical Analysis

Statistical analysis was performed using Prism software (GraphPad Prism6.0). Statistically significant differences were assessed by either unpaired *t*-test or one-way ANOVA or two -way ANOVA with Bonferroni's multiple comparison test. Log-rank (Mantel-Cox) test was used to analyze mouse survival curves. Bars in all graphs are expressed as mean ± SEM. “ns” denotes no significance. Significance was marked by asterisk (^*^P < 0.05; ^**^P < 0.01; ^***^P < 0.001; and ^****^P < 0.0001).

## Results

### NIH3T3-CM Enhances Effector Functions of CD8^+^ CTLs

**Figure 1:**
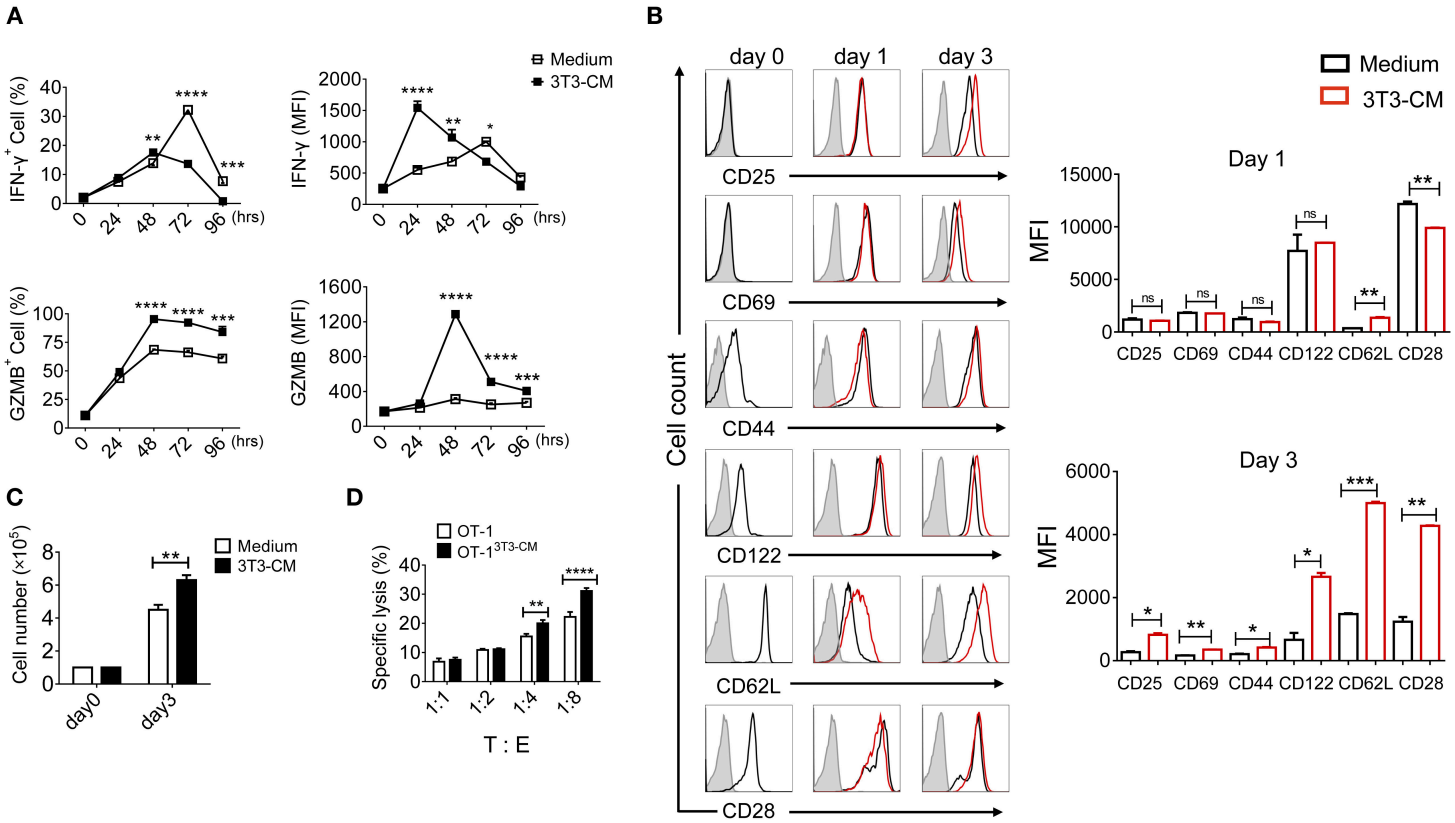
NIH3T3-CM strengthens effector functions of CTLs. OT-1 CD8^+^ T cells were stimulated with K^b^-OVA beads in the presence or absence of NIH3T3-CM (50%, v/v). **(A)** Intracellular flow cytometric determination of expression levels of IFN-γ and granzyme B (GZMB) at indicated time points. Frequencies of IFN-γ or GZMB positive CD8^+^ T cells are shown (left panel). MFI (mean fluorescence intensity) of IFN-γ or GZMB was plotted for IFN-γ^+^ or GZMB^+^ cells (right panel). **(B)** Levels of surface molecules were determined by flow cytometry at indicated time points. Left, representative histograms. Right, MFI values. The gray histogram as a background represents cells that are not stained. **(C)** Cell numbers were counted after 3 days of stimulation. **(D)** Specific cytotoxicity of OT-1 CTLs generated in the presence or absence of NIH3T3-CM against EG.7 cells was evaluated at the indicated effector: target (E:T) ratios. All data are representatives of at least three independent experiments. Data are presented as mean ± SEM; *p < 0.05, **p < 0.01, ***p < 0.001, and ****p < 0.0001. Two-way ANOVA with Bonferroni's multiple comparison tests.

Fibroblasts with different subsets are functionally heterogeneous (either stimulatory or inhibitory) on T lymphocytes (24). Our previous report has shown that MEFs can significantly enhance effector functions of CTLs through up-regulation of IFN-γ and granzyme B expression (19). Therefore, we determined whether NIH3T3 enhanced effector functions of CTLs upon TCR stimulation. Co-culturing CD8^+^ T cells with NIH3T3 using trans-wells showed that NIH3T3-enhanced IFN-γ and granzyme B expressions of CD8^+^ T cells were independent of direct contact (Supplementary Figure 1). Thus, NIH3T3-CM was focused. To further analyze the impact of NIH3T3-CM on CD8^+^ T cells during priming, kinetic patterns of IFN-γ and granzyme B productions were evaluated (Figure 1A). We found that NIH3T3-CM strongly promoted antigen induced IFN-γ expression in an acute manner within 24 h. The level of IFN-γ peaked at 24 h and then declined thereafter. It seemed that NIH3T3-CM could expedite kinetic IFN-γ production of CTLs compared to that of medium alone-cultured CTLs which peaked at 72 h (Figure 1A, top panel). Contrast to acute enhancement in IFN-γ production, NIH3T3-CM continuously enhanced granzyme B expression. The level of granzyme B in NIH3T3-CM-cultured CTLs peaked at 48 h after TCR stimulation and declined thereafter. The frequency of granzyme B producing CTLs peaked at 48 h in the condition of medium alone culturing while granzyme B levels per cell fluctuated modestly (Figure 1A, bottom panel). Next, the expressions of typical surface molecules associated with T cell differentiation in NIH3T3-CM-cultured CTLs were compared with those in medium-alone-cultured CTLs. After 3 days of TCR stimulation, expressions of CD25 (IL2Rα), common β receptor CD122 (IL-2β, IL-15β), and CD28 were increased significantly while expressions of CD69 (early inducible activation marker) and CD44 increased modestly (Figure 1B). The level of “lymph homing receptor” CD62L (L-selectin), a naïve and central memory marker, was decreased on day 1 after TCR stimulation. It was then up-regulated thereafter. Surprisingly, the level of CD62L was much higher on NIH3T3-CM-cultured CTLs (Figure 1B). We also examined whether NIH3T3-CM could enhance the proliferative capacity of CTLs. After 3 days of stimulation, cell numbers of NIH3T3-CM-cultured CTLs were higher than those of medium-cultured CTLs (Figure 1C). Enhanced cytotoxic activity was also observed in NIH3T3-CM-cultured CTLs (Figure 1D). Taken together, these results suggest that NIH3T3-CM can strengthen effector functions of CTLs following antigen priming.

### NIH3T3-CM Improves the Intrinsic Quality of CTLs Which May Acquire Memory-Fated Potential

**Figure 2:**
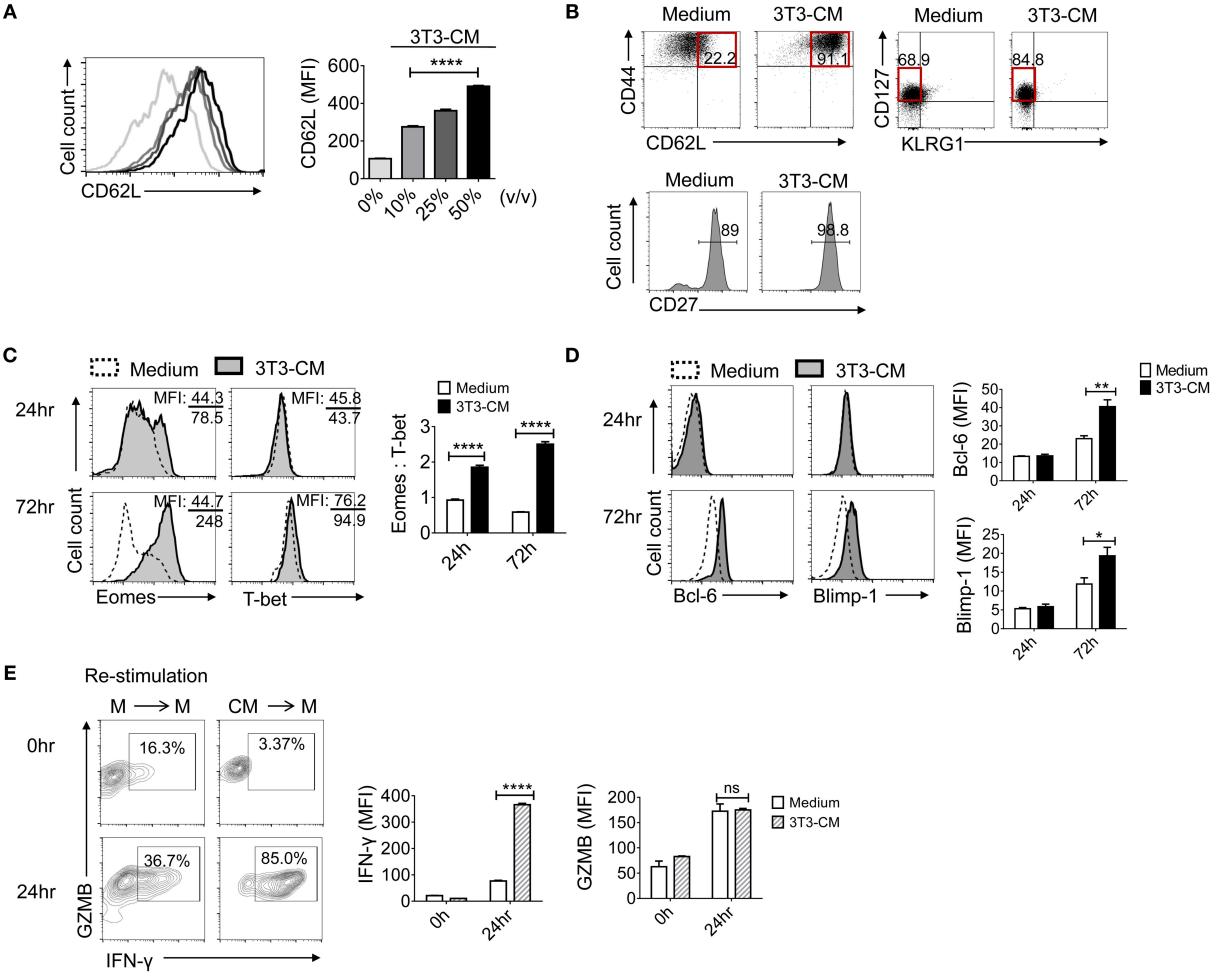
NIH3T3-CM confers potential of memory cell differentiation to CTLs**. (A)** Sorted OT-1 CD8^+^ cells were stimulated with different volumes of NIH3T3-CM and CD62L levels on cell surface were measured on day 3. **(B)** Typical surface markers representing central memory cells and MPECs were evaluated after 3 days of stimulation. **(C,D)** Transcriptional factors were examined by intracellular FACS analysis after 24 h and 72 h of stimulation. Representative FACS data are shown (**C**, left). The ratio of Eomes to T-bet was calculated by their MFI (**C**, right). Representative FACS data are shown (**D**, left). Quantification of Bcl-6 and Blimp-1 was plotted respectively (**D**, right). **(E)** OT-1 CD8^+^ cells were stimulated in the presence or absence of NIH3T3-CM for 4 days. CTLs were then washed and re-stimulated in the absence of NIH3T3-CM for another 24 h followed by intracellular FACS analysis. M, RPMI-1640 medium; CM, NIH3T3-CM. Representative FACS data are shown (left panel). Quantification of IFN-γ and GZMB was plotted respectively (right panel). All data are representatives of at least three independent experiments. Data are presented as mean ± SEM; *p < 0.05, **p < 0.01, ****p < 0.0001. One-way **(A)** or Two-way **(C–E)** ANOVA with Bonferroni's multiple comparison tests.

As shown in Figure 1B, CD62L was expressed much higher on NIH3T3-CM-cultured CD8^+^ T cells. Prompted by this result, we investigated whether NIH3T3-CM directed the differentiation of antigen-primed CD8^+^ T cells toward memory type cells. First, we determined whether NIH3T3-CM might have dose-dependent effects on surface expression level of CD62L on CTLs. Results showed that expression levels of CD62L were directly correlated with treated amount of NIH3T3-CM (Figure 2A). Consequently, higher frequency of CD44^++^CD62L^++^ population representing the phenotype of central memory cells was observed in NIH3T3-CM-cultured CTLs (Figure 2B). Consequently, NIH3T3-CM enhanced effector functions of CTLs along with enhancement of central memory markers such as CD44, CD62L (Figure 2B), and CD122 (Figure 1B). In this context, we questioned whether NIH3T3-CM-programmed CTLs were memory-fated effector clones. Accumulating evidences have established that T cells can integrate modulated signals during priming to determine their fate (MPECs or SLECs) (10, 12, 13). Hence, we investigated whether NIH3T3-CM-cultured CTLs were also phenotypically consistent with MPECs [CD27^++^CD127(IL-7Rα)^++^KLRG1 (markers of senescent CD8^+^T cells)^−^]. Phenotypic analysis showed that NIH3T3-CM-cultured CTLs displaying CD27^++^CD127^+/−−^KLRG1^−^ were partially in line with MPECs. However, NIH3T3-CM did not significantly regulate expression of CD127, CD27, or KLRG1 as their expression levels were comparable with those of medium alone-cultured CTLs (Figure 2B). Collectively, these results suggest that high level of central memory markers (CD44 CD62L and CD122) and high level of CD27 as well as intermediate level of CD127 might give NIH3T3-CM-cultured CTLs potential in differentiation of memory cells, although the phenotype of NIH3T3-CM-cultured CTLs is not totally consistent with what is generally considered MPECs.

Next, we explored whether NIH3T3-CM influenced transcriptional programming in CD8^+^ T cells following antigen priming. T-box transcription factors, T-bet, and eomesodermin (Eomes) play essential roles in inducing CD8^+^ T cells acquisition of effector functions and formation of memory pools (25–28). To investigate whether NIH3T3-derived factor(s) influenced the expression of T-box transcription factors, we examined expression levels of T-bet and Eomes during 72 h of TCR stimulation (Figure 2C). After 72 h of stimulation, NIH3T3-CM strongly induced CTLs expression of Eomes. In striking contrast, medium alone-cultured CTLs expressed Eomes at low levels without showing significant difference between 24 h and 72 h of stimulation. Compared to strongly induced Eomes by NIH3T3-CM in CTLs, there was no significant enhancement of T-bet level in response to NIH3T3-CM treatment. Given the notion that Eomes expression appears to preferentially increase relative to T-bet in memory cell differentiation and homeostasis (12, 25, 28, 29), we performed analysis of mean fluorescence intensity (MFI) for Eomes and T-bet. Results showed that NIH3T3-CM-cultured CTLs had much higher ratio of Eomes to T-bet compared to medium cultured CTLs (Figure 2C, right panel). These data indicate that Eomes might play a role in NIH3T3-CM-induced enhancement of effector function as well as in conferring potentials of memory cell differentiation. To investigate whether NIH3T3-CM regulated other transcriptional factors involved in the control of memory cell differentiation, we analyzed another pair of antagonistic transcription factors: Bcl-6 and Blimp-1. It has been well-characterized that Bcl-6 controls memory cell differentiation while Blimp-1 is crucial for effector function acquisition, including cell proliferation, cytotoxicity, and cytokine production (30–33). After 72 h of TCR stimulation, NIH3T3-CM slightly enhanced levels of Bcl-6 and Blimp-1 (Figure 2D). Collectively, these results indicate that NIH3T3-CM can affect intrinsic transcriptional events of CTLs which helps differentiation, effector function, and memory development of CTLs.

Vigorous response of CTLs after re-encountering antigens reflects the potential of CTLs in memory cell differentiation. Therefore, we examined the re-stimulatory activity of CTLs programmed by NIH3T3-CM following initial priming (Figure 2E). The production level of granzyme B was comparable between the two different pre-cultured T cells. However, NIH3T3-CM-educated CTLs dramatically produced higher level of IFN-γ than medium alone-cultured CTLs after re-encountering antigens (Figure 2E, right). Consequently, high frequency of IFN-γ^+^ granzyme B^+^ cells were obtained in NIH3T3-CM-educated CTLs (Figure 2E, left).

Based on these findings, we conclude that CTLs derived with NIH3T3-CM displaying a phenotype of CD44^++^CD62L^++^CD122^++^CD25^++^CD27^++^CD127^+/−^ and KLRG1^−^ as well as transcriptional program of Eomes^++^T-bet^++^Bcl-6^+^Blimp-1^+^ possess high effector function and vigorous re-stimulatory activity. These characteristics implicate that NIH3T3-CM can enhance intrinsic properties of CTLs and direct development of memory precursors following antigen priming.

### NIH3T3-CM Educated CTLs Can Develop to Long-Lived Memory Cells

**Figure 3:**
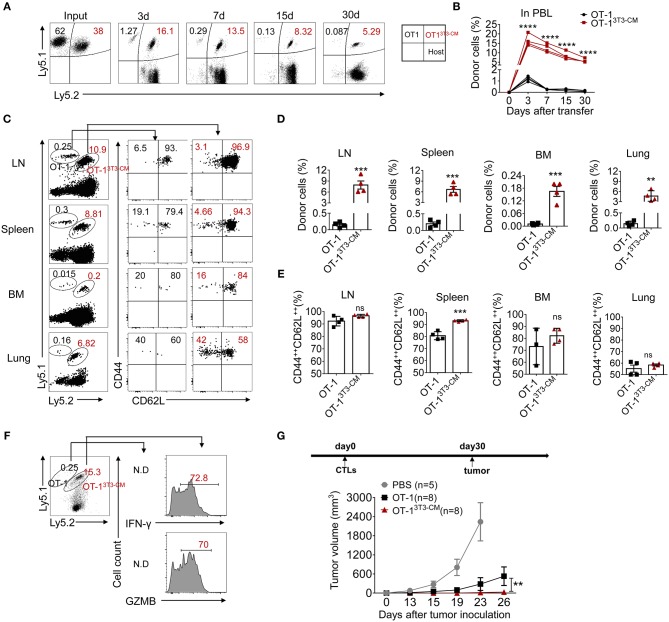
NIH3T3-CM-educated CTLs develop into functional central memory cells. **(A–F)** Congenic OT-1 CTLs were obtained by 3 days activation in the presence or absence of NIH3T3-CM. 2.4 × 10^6^ Ly5.1^+/+^OT-1 CTLs (RPMI-1640 medium) and 1.6 × 10^6^ Ly5.1^+^ Ly5.2^+^ OT-1 CTLs (NIH3T3-CM, 50% v/v) with ratio of 6:4 were i.v. co-transferred to WT C57BL6/c mice (Ly5.2^+/+^, *n* = 4). **(A,B)** Frequencies of transferred cells in peripheral blood were determined at indicated time points. **(A)** Representative FACS data show frequencies of donor cells. **(B)** Graphs show the summary of dot plots of **(A)** with each dot representing for a recipient. **(C–E)** Frequencies of transferred OT-1 cells that migrated to spleen, inguinal lymph node (LN), bone marrow (BM), and lung were evaluated after 30 days of T cell transfer. The phenotype of transferred cells that displayed central memory cells (CD44^++^CD62L^++^) and effector memory cells (CD44^++^CD62L^−^) was evaluated. **(C)** Representative FACS data are shown. Histograms represent frequencies of transferred cells **(D)** and frequencies of central memory cells in transferred cells **(E)**. Each dot represents a single mouse. **(F)** Whole splenocytes were re-stimulated with OVA_257−264_ peptides for 2 days *in vitro*. Levels of IFN-γ and GZMB were evaluated by intracellular FACS analysis. Representative FACS data are shown. **(G)** Effector OT-1 cells (1 × 10^6^/mouse) generated in the presence or absence of NIH3T3-CM were transferred to WT C57BL6/c mice. One month later, EG.7 tumor cells (2 × 10^5^/mouse) were s.c inoculated to mice. Tumor growth was monitored. PBS group (*n* = 5), OT-1 group (*n* = 8), OT-1^3T3−CM^ group (*n* = 8). All data are representatives of two independent experiments. Data are presented as mean ± SEM; **p < 0.01, ***p < 0.001, and ****p < 0.0001. Two-way ANOVA with Bonferroni's multiple comparison tests **(A, G)**; Unpaired *t*-test **(A,E)**.

To determine whether NIH3T3-CM-programmed effector clones with memory characteristics could further differentiate to long-lived memory cells, we co-transferred both NIH3T3-CM-educated OT-1 CTLs and medium alone-cultured OT-1 CTLs into WT B6 recipients and analyzed the persistence potential of infused cells using their congenic markers Ly5.1 and Ly5.2. Kinetic frequencies of transferred cells in peripheral blood were detected. During 30 days of examination, frequencies of NIH3T3-CM-educated CTLs showed gradual decline. In striking contrast, frequencies of medium alone-cultured CTLs showed rapid decline (Figures 3A,B). Consequently, very low number of medium alone-cultured CTLs survived in peripheral blood whereas higher frequency of NIH3T3-CM-cultured CTLs persisted until 30 days after cell transfer (Figures 3A,B). The persistence of co-transferred OT-1 cells in other tissues was also analyzed. Consistent with the higher frequency of NIH3T3-CM-educated CTLs that persisted in blood, much higher frequencies of NIH3T3-CM-educated CTLs also homed to lymphoid organs (lymph node, spleen and bone marrow) and non-lymphoid organ (lung) compared to those of medium alone-cultured CTLs (Figures 3C,D). Phenotypic analysis showed that the majority of persisted cells displayed central memory markers (CD44^++^CD62L^++^) (Figures 3C,E). To determine the stimulatory activity of memory cells, whole splenocytes were re-stimulated *in vitro*. In response to OVA_257−264_ peptide stimulation, we observed high levels of IFN-γ and granzyme B productions in memory cells derived from NIH3T3-CM-educated OT-1 cells. The number of memory OT-1 cells deriving from control medium-cultured CTLs was below detectable level (Figure 3F). The hallmark of memory CD8^+^ T cells is their ability to generate protective immune response when re-exposed to antigens (34). To further assess the anti-tumor immunity of memory CD8^+^ T cells derived from NIH3T3-CM-educated CTLs responding to tumors, OT-1 CTLs generated in the presence or absence of NIH3T3-CM were transferred to B6 WT mice. One month later, large dose of tumor cells were inoculated to mice. It was observed that tumor growth was further inhibited in the group that received NIH3T3-CM-educated OT-1 cells compared to the group received medium alone-cultured OT-1 cells (Figure 3G). Taken together, these two adoptive transfer experiments demonstrated that NIH3T3-CM-programmed CTLs had high potential to develop into long-lived memory cells which could efficiently protect mice from tumor growth.

### NIH3T3-CM-Educated CTLs Regress Tumor Growth Effectively

**Figure 4:**
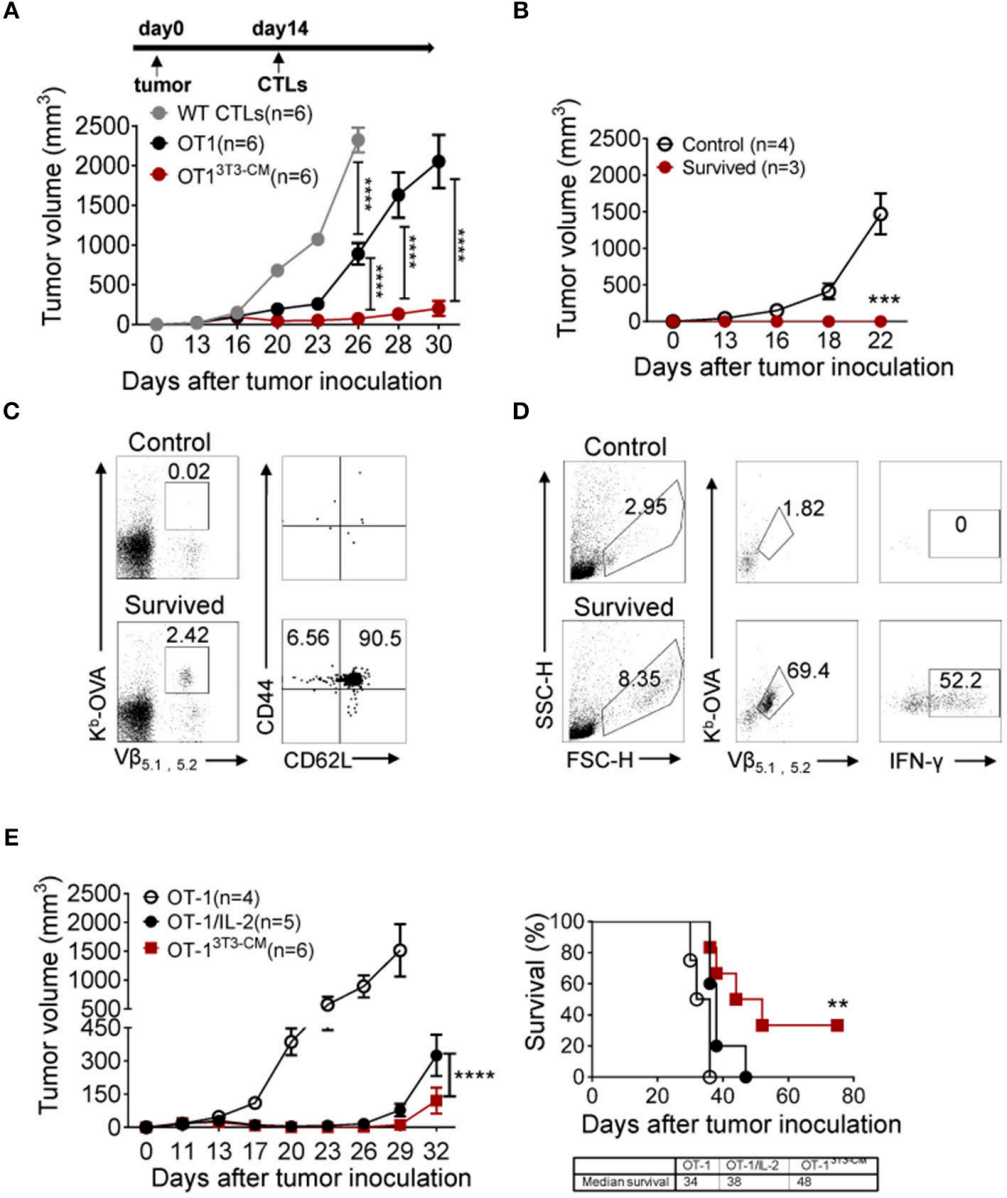
NIH3T3-CM-educated OT-1 CTLs effectively regress EG.7 tumor growth along with memory cell generation. **(A)** EG.7 tumor cells (5–7 × 10^4^/mouse) were s.c. inoculated to WT C57BL6/c mice. Then 30–50 mm^3^ tumor was established (usually 12–14 days after tumor inoculation). WT CTLs (anti-CD3/CD28 stimulation), medium alone, or NIH3T3-CM cultured OT-1 CTLs (K^b^/OVA beads stimulation, 1 × 10^6^/mouse) were i.v. injected into tumor-bearing mice (*n* = 6/group). Tumor growth was then monitored. Serial tumor measurements were obtained. **(B)** At day 60 after the first tumor challenge, after removing mice that showed any sign of tumor growth, survived mice that have received NIH3T3-CM-cultured OT-1 CTLs were re-challenged with E.G7 tumor cells (5–7 × 10^4^/mouse). No tumor experienced mice as control also received the same dose of tumor cells. Tumor growth was then monitored. **(C)** At 22 days after tumor re-challenge, mice were sacrificed and tumor specific CD8^+^ T cells in spleen were analyzed. K^b^-OVA tetramer staining was used to detect tumor specific CD8^+^ T cells. Memory phenotype of tumor specific T cells was analyzed by anti-CD44 and anti-CD62L antibody. **(D)** Whole splenocytes were re-stimulated with OVA_257−264_ peptides for 2 days *in vitro*. Activated antigen specific T cells and the expression of IFN-γ in antigen specific T cells were determined. **(E)** As described in A, after 12 days of tumor inoculation, OT-1 CTLs were transferred to tumor-bearing mice. For the group of IL-2 administration, 2 μg IL-2 once on the day of CTL injection and twice a day on the two following days were i.p. injected to mice. Tumor growth and survivals were monitored (*n* = 4 in OT-1 group; *n* = 5 in OT-1/IL-2 group; *n* = 6 in OT-1^3T3−CM^ group). Data are representatives of three independent experiments **(A)** or two experiments **(B–E)**. Error bars indicate SEM. **P < 0.01; ***P < 0.001; ****P < 0.0001. Two-way ANOVA with Bonferroni's multiple comparison tests; Comparison of survived curves with Log-rank (Mantel-Cox) tests.

Long-term persistence and function of transferred cells are crucial factors in ACT for cancers (4, 5). Therefore, we assessed therapeutic effects of NIH3T3-CM-educated CTLs using an established tumor model. *In vitro*-activated OT-1 cells generated in the presence or absence of NIH3T3-CM were respectively, transferred into WT B6 mice bearing EG.7 tumors. As expected, NIH3T3-CM-educated OT-1 CTLs dramatically regressed tumor growth (Figure 4A). Because timely preparation of sufficient number of *in vitro*-expanded CTLs with high functionality is a limitation for ACT (5), antitumor efficiencies of NIH3T3-CM-educated CTLs with serially diluted numbers were compared with those of determined number of medium alone-cultured CTLs. Results showed that NIH3T3-CM-educated CTLs effectively regressed tumor growth even at a quarter number of medium alone-cultured CTLs (Supplementary Figure 2). This implies that NIH3T3-CM-educated CTLs can have therapeutic benefit even with a limited number of CTLs for ACT. To determine whether survived mice could maintain tumor reactive memory OT-1 cells, the same dose of EG.7 tumor cells was used for re-challenge. Tumor growth was observed in no tumor-experienced mice. However, tumors failed to be established on first-round survivors (Figure 4B). In the spleen of survivors, significant numbers (> 2% of all lymphocytes) of tumor reactive OT-1 cells were detected. Most of them displayed a central memory phenotype (Figure 4C). The functionality of these maintained OT-1 cells was further confirmed by measuring IFN-γ production level after *in vitro* re-stimulation (Figure 4D). These data demonstrate that NIH3T3-CM-educated CTLs not only exert superior antitumor effects, but also establish long-term protective immunity to prevent tumor relapse.

Adoptive T cell therapy together with exogenous IL-2 administration is considered a relatively effective method that has been extensively used in clinical trials (35). Despite clinical successes, IL-2 treatment has fatal defect in that high dose of IL-2 administration can induce severe dose-limiting toxicities in patients (35, 36). In this regard, we compared the therapeutic effect of NIH3T3-CM-educated CTLs with that of medium alone-cultured CTLs with co-administration of exogenous IL-2 (Figure 4E). As expected, co-administration of IL-2 significantly improved the antitumor activity of medium alone-cultured OT-1 cells. However, it exerted lower antitumor effects compared to adoptive transfer of NIH3T3-CM-educated OT-1 cells without IL-2 co-administration (Figure 4E, left). In consistence with regressed tumor growth, NIH3T3-CM-educated OT-1 cells prolonged mice survival than medium alone-cultured OT-1 cells with concomitant IL-2 therapy (Figure 4E, right).

**Figure 5:**
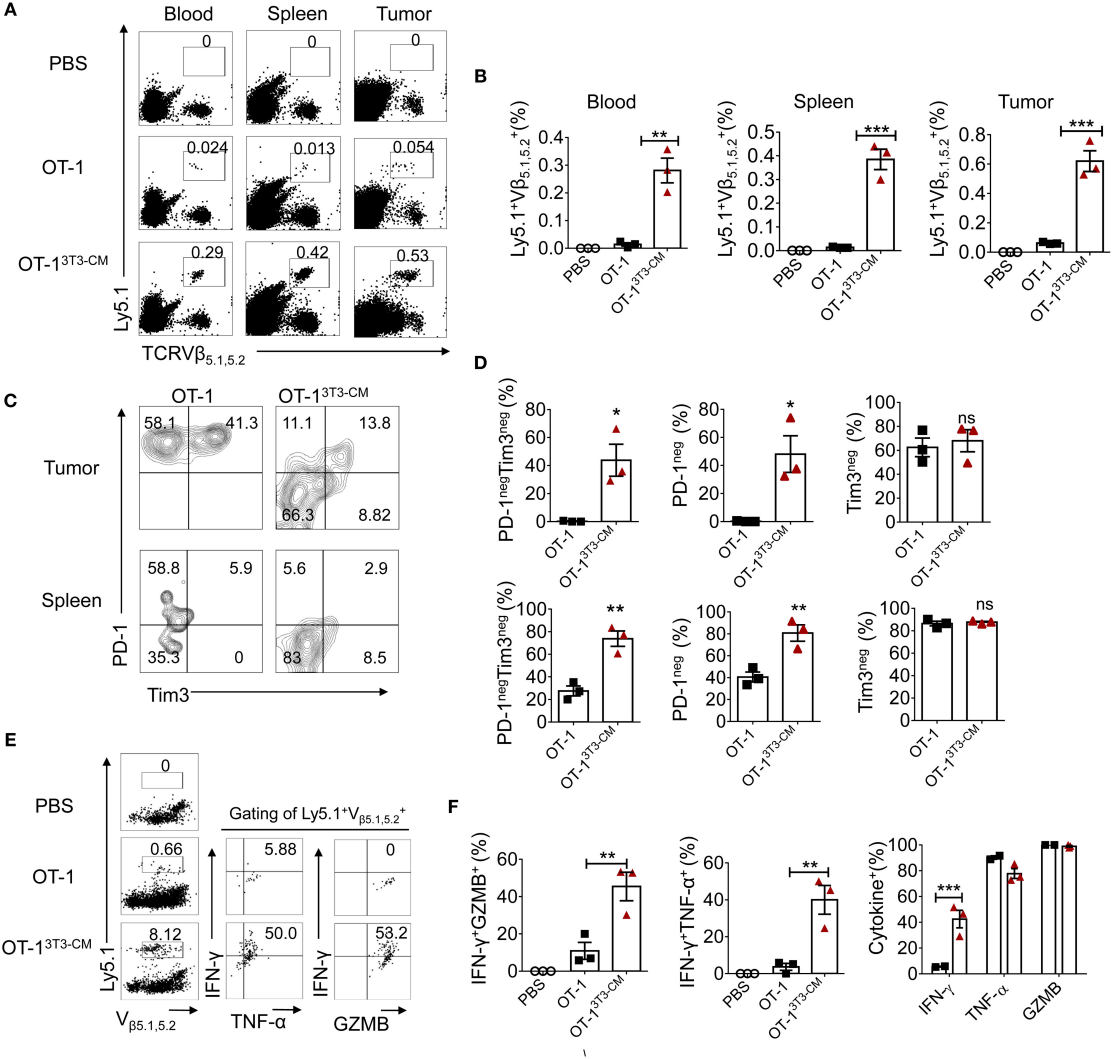
High persistence and low PD-1 expression of NIH3T3-CM-educated OT-1 CTLs in tumor-bearing mice. 1 × 10^5^ EG.7 tumor cells were s.c inoculated to WT C57BL6/c (Ly5.2^+/+^) mice (1 × 10^5^/mouse). After tumor growth reached 50~70 mm^3^, 1 × 10^6^ congenic Ly5.1^+/−^ OT-1 CTLs generated in the presence or absence of NIH3T3-CM (*n* = 3/group) were i.v transferred to tumor-bearing mice. **(A,B)** Frequencies of transferred Ly5.1^+^ OT-1 cells in blood, spleen, and tumor were analyzed at 6 days after T cell transfer. **(A)** Representative FACS data are shown. **(B)** Summarized results of **(A)**. Each dot represents a single mouse. **(C–D)** FACS analysis of expression level of PD-1 and Tim3 on Ly5.1^+^ OT-1 cells in tumor and spleen. **(C)** Representative FACS data and **(D)** summarized frequencies of populations from **(C)** are shown. Each dot represents a single mouse. **(E,F)** Whole splenocytes were treated with OVA_257−264_ peptides *in vitro* for 2 days. Expression levels of IFN-γ, TNF-α, and GZMB were evaluated by intracellular FACS analysis. **(E)** Representative FACS data and **(F)** summarized frequencies of cytokine positive cells from **(E)** are shown. Each dot represents a single mouse. Two independent experiments were carried out and similar results were obtained. Data are presented as mean ± SEM *P < 0.05; **P < 0.01; ***P < 0.001. Two-way ANOVA with Bonferroni's multiple comparison tests **(B,F)**; Unpaired *t*-test **(D)**.

As superior antitumor immunity was observed in the group of NIH3T3-CM-educated CTLs treatment, we examined the frequency of transferred cells in tissues of tumor-bearing mice. In line with efficient tumor regression by NIH3T3-CM-educated CTLs, significantly higher frequencies of NIH3T3-CM-educated CTLs persisted in peripheral blood, spleen, and tumor site in comparison with those of medium alone-cultured CTLs (Figures 5A,B). Because tumor cell-mediated exhaustion of CTLs could affect the number and overall functionality of tumor-specific CTLs, we questioned whether NIH-3T3-education could affect expressions of immune check point molecules on CTLs. Thus, we analyzed expression levels of PD-1 and Tim-3 on NIH3T3-CM-educated CTLs or medium alone-cultured CTLs that migrated to tumors. Results showed that about half frequency of NIH3T3-CM-educated CTLs did not express PD-1 whereas nearly all of medium alone cultured-CTLs expressed high level of PD-1 (Figures 5C,D, top panel). Expression levels of another exhaustion marker Tim3 were comparable between these two cultures. In consistent with patterns of PD-1 and Tim3 expression levels observed on TILs, down-regulation of PD-1 expression and comparable levels of Tim3 expression were also found on NIH3T3-CM educated CTLs that migrated to the spleen (Figures 5C,D, bottom panel). Next, effector function of transferred cells was compared between the two groups through re-stimulation *in vitro*. We observed that NIH-3T3-educated CTLs displaying lower level of PD-1 expression significantly increased IFN-γ production (Figures 5E,F). Expression levels of TNF-α and granzyme B were comparable between the two groups. This implies that increased IFN-γ production of PD-1^low^ CTLs might enhance inflammatory response in tumor tissues. Taken together, these results demonstrate that NIH3T3-CM-educated CTLs exhibiting low PD-1 expression exert superior persistence and function, thus contributing to their strong antitumor effects following transfusion.

### NIH3T3-CM-Programmed CTLs Express Low Levels of PD-1

**Figure 6:**
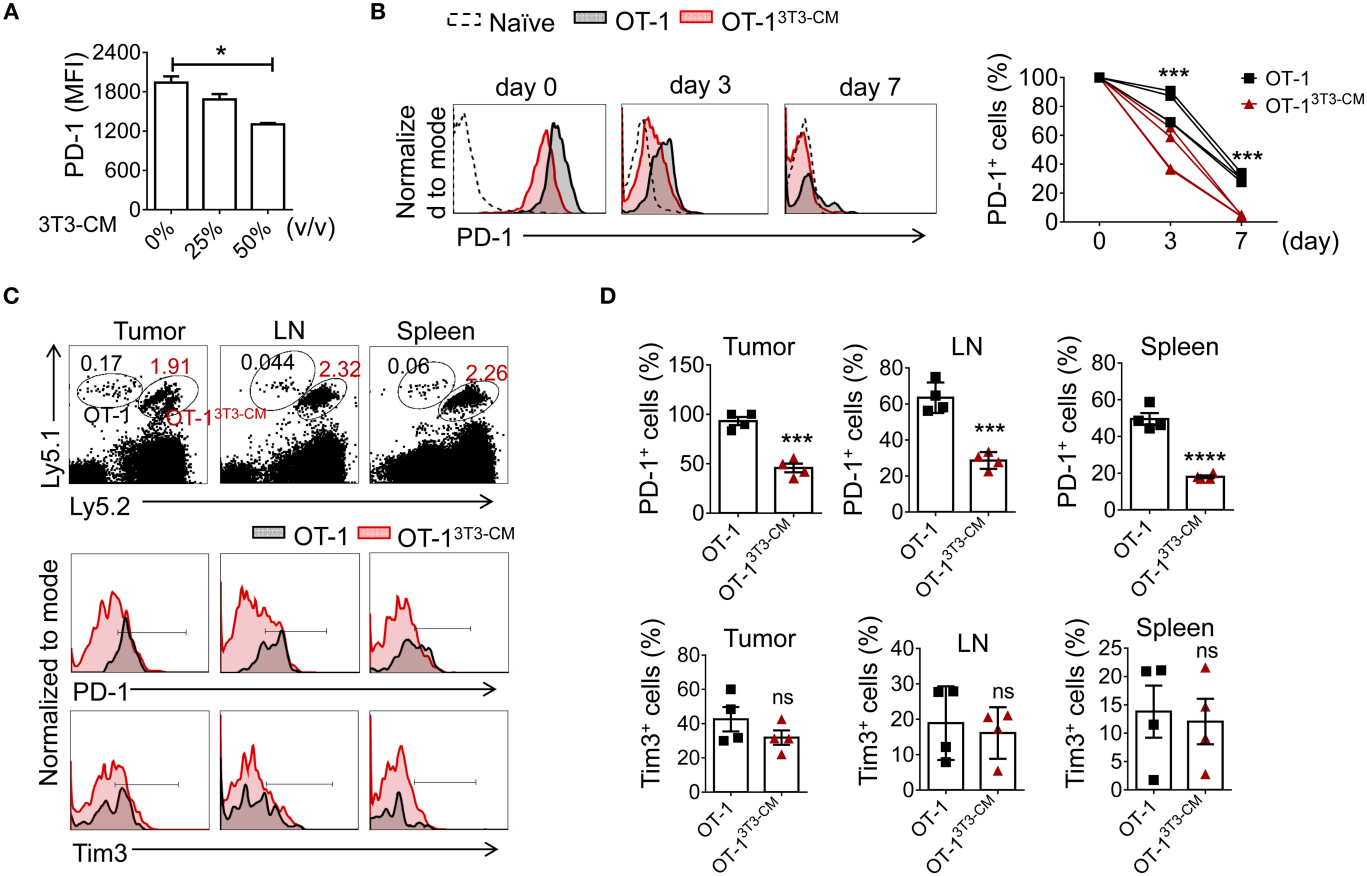
NIH3T3-CM -programmed CTLs intrinsically express low levels of PD-1. **(A)** OT-1 CD8^+^T cells were stimulated with different volumes of NIH3T3-CM for 3 days and levels of PD-1 on CTLs were analyzed. **(B–D)** Congenic OT-1 CTLs [Ly5.1^+/+^ OT-1 cells cultured in medium alone, Ly5.1^+^ Ly5.2^+^ OT-1 cells cultured in NIH3T3-CM (50%, v/v)] were co-transferred with ratio of 1:1 to **(B)** naïve WT C57BL6/c (Ly5.2^+/+^) mice (*n* = 4) or **(C**,**D)** EG.7 tumor (50~100 mm^3^)-bearing WT C57BL6/c (Ly5.2^+/+^) mice (*n* = 4). **(B)** Levels of PD-1 on transferred cells in peripheral blood lymphocytes (PBL) of naive mice were analyzed at indicated time points using FACS analysis. **(C,D)** Levels of PD-1 and Tim3 on transferred cells in tumor mass, inguinal lymph node, and spleen of tumor-bearing mice were analyzed at 6 days after T cell transfer. **(C)** Representative FACS analysis and **(D)** frequencies of PD-1^+^ cells or Tim3^+^ cells in transferred cells are shown. Each dot represents a mouse. Data are representatives of three independent experiments **(A)** or two experiments **(B–D)**. Data are presented as mean ± SEM. *P < 0.05; ***P < 0.001; ****P < 0.0001. One-way **(A)** or Two-way **(B)** ANOVA with Bonferroni's multiple comparison tests; Unpaired *t*-test **(D)**.

In Figures 5C,D, we observed that NIH3T3-CM-educated CTLs expressed low levels of PD-1 in both tumor and spleen. Therefore, we wondered whether the decreased PD-1 expression might be an intrinsic characteristic of NIH3T3-CM-programmed CTLs. We first stimulated OT-1 cells *in vitro* in the presence of different volumes of NIH3T3-CM and determined PD-1 expression. Results showed that NIH3T3-CM down-regulated CTLs expression of PD-1 during antigen priming in a dose-dependent manner (Figure 6A). It is known that PD-1 is transiently expressed on T cells after priming and down-regulated after antigen clearance (37). Thus, we assessed whether PD-1 expression on CTLs was down-regulated after transfer into antigen-free mice (Figure 6B). Without antigen exposure, both medium alone and NIH3T3-CM-cultured CTLs decreased PD-1 expression. After 7 days of cell transfer, most of NIH3T3-CM-educated CTLs abrogated PD-1 expression whereas a certain number of medium alone-cultured CTLs still maintained PD-1 expression (Figure 6B). No significant increase in Tim3 level was observed on both cultured CTLs (data not shown). Because NIH3T3-CM-educated CTLs exhibited more potent ability of tumor rejection, the tumor microenvironment of the mouse that received NIH3T3-CM-educated CTLs might have established more inflammatory status compared to that of mouse that received medium alone-cultured CTLs. Difference in tumor microenvironment might also affect expression levels of PD-1 on CTLs surrounded by such environment. To test whether the relatively lower level of PD-1 expression on NIH3T3-CM-educated TILs shown in Figures 5C,D was influenced *in situ* or was an intrinsic characteristic conferred by NIH3T3-CM during *in vitro* culture, we co-transferred these two cultured OT-1 CTLs to EG.7 tumor-bearing mice (Figures 6C,D). After 6 days of T cell transfer, transferred OT-1 cells in tumor, lymph node, and spleen were analyzed. We found that NIH3T3-CM-educated OT-1 (Ly5.1^+^Ly5.2^+^) expressed low levels of PD-1 whereas medium alone-cultured OT-1 (Ly5.1^+/+^) expressed high levels of PD-1 regardless of analyzed tissues (Figures 6C,D, top panel). Different from results of PD-1 expression, expression pattern of Tim3 was comparable between the two cultures (Figures 6C,D, bottom panel). Taken together, these results suggest that low PD-1 expression is an intrinsic characteristic of NIH3T3-CM-programmed CTLs.

### NIH3T3-CM Confers Potential of Human Memory CD8^+^ T Cell Differentiation

**Figure 7:**
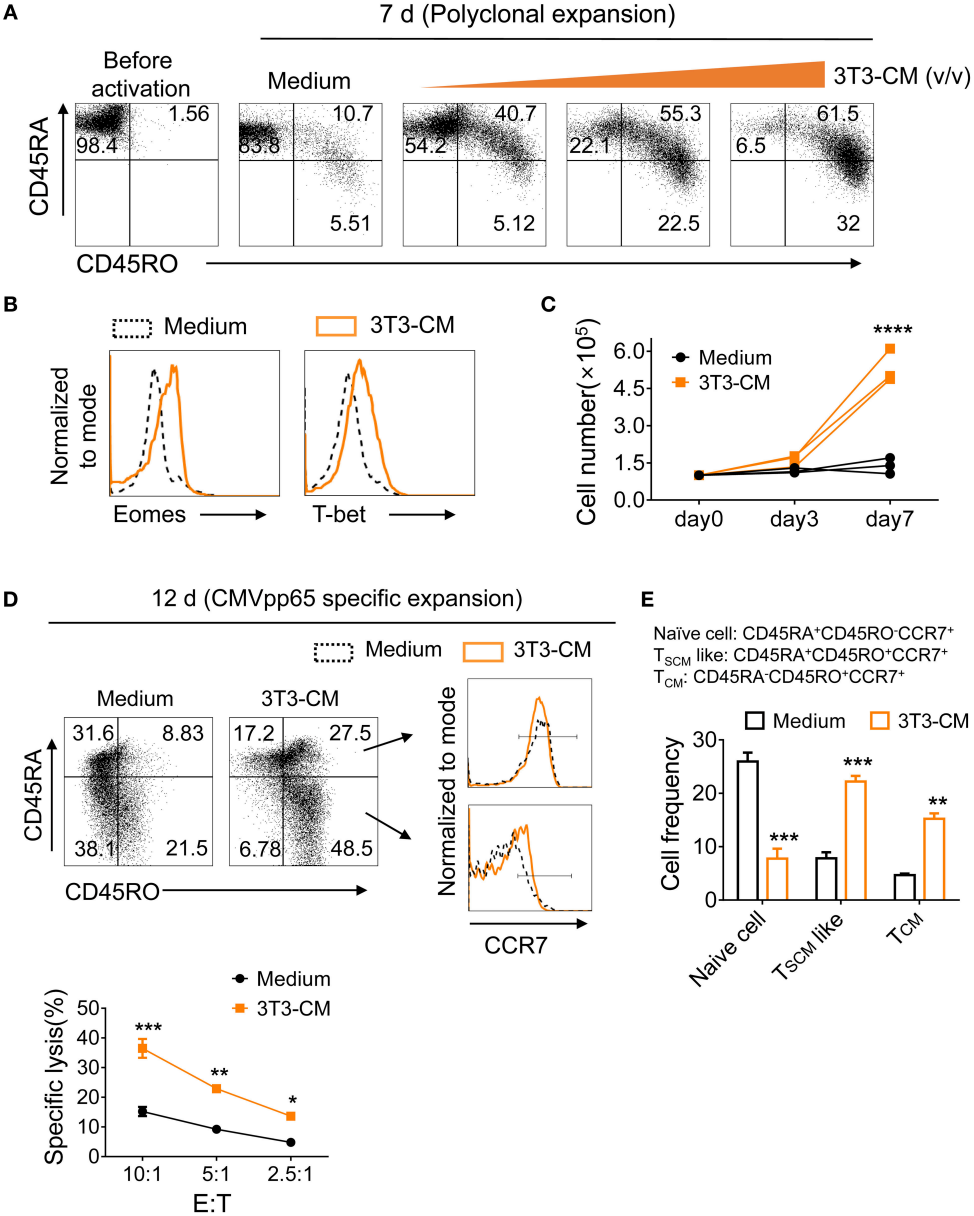
NIH3T3-CM confers potential of human memory CD8^+^ T cell differentiation. **(A–C)** Purified human naïve CD8^+^ T cells were expanded by OKT3/anti-CD28 in the absence or presence of increased volumes of NIH3T3-CM. After 7 days of expansion, surface phenotypic changes **(A)** and transcription factors **(B)** were analyzed by FACS analysis. **(C)** Counted cell numbers at indicated time points. Each line in the same color represents a donor. **(D–F)** Purified human naïve CD8^+^ T cells were expanded by CMVpp65-peptide-loaded PBMCs (irradiated) in the presence or absence of NIH3T3-CM. After 12 days of expansion, surface phenotype of CD8^+^ T cells **(D)**, frequencies of naïve and memory CD8^+^T cells **(E)**, and specific cytotoxicity of the expanded CD8^+^ T cells **(F)** were determined. Representative results of three donors. Data are presented as mean ± SEM. *P < 0.05; **P < 0.01; ***P < 0.001; ****P < 0.0001. Two-way ANOVA with Bonferroni's multiple comparison tests **(C,E,F)**.

To determine whether murein-derived NIH3T3-CM can also have the same effect on human CD8^+^ T cells, naïve CD8^+^ T cells isolated from peripheral blood lymphocytes (PBL) were polyclonally expanded with increasing amount of NIH3T3-CM and surface expression levels of markers CD45RA (naïve T cells) and CD45RO (antigen experienced T cells) were analyzed. We observed that NIH3T3-CM increased CTLs expression of CD45RO along with decrease of CD45RA expression (Figure 7A). Levels of T-box transcription factors, T-bet, and Eomes were upregulated by NIH3T3-CM (Figure 7B). Numbers of CTLs expanded by anti-CD3 antibody were also dramatically increased when NIH3T3-CM was treated (Figure 7C). To test their effects on antigen-specific clones, human naïve CD8^+^ T cells were expanded by CMV-pp65 peptide-loaded PBMC in the presence or absence of NIH3T3-CM. After 12 days of expansion, NIH3T3-CM significantly enhanced levels of CD45RO, consistent with conditions of polyclonal expansion (Figure 7D). Higher frequency of antigen experienced clones with similar phenotype of memory stem T cells (CD45RA^+^CD45RO^+^CCR7^+^) was obtained in NIH3T3-CM-cultured CTLs (Figures 7D,E). NIH3T3-CM also elevated CCR7 expression in CD45RA^−^CD45RO^+^ cells which dramatically enhanced the frequency of central memory phenotypic cells (CD45RA^−^CD45RO^+^CCR7^+^) (Figures 7D,E). These results suggest that NIH3T3-CM not only confers memory characters of mouse CD8^+^ T cells, but also modulates differentiation of human memory CD8^+^ T cells. To anticipate tumor reactivity of NIH3T3-CM-educated human CTLs with memory characters, their cytotoxic activities were determined. NIH3T3-CM-educated CTLs had higher cytotoxicity than medium alone-cultured CTLs (Figure 7F). Mouse embryonic fibroblasts (MEFs) can provide various growth factors and suitable attachment substrates. They are widely used as feeders to support human embryonic stem cell self-renewal and growth (38). Mouse 3T3 cells also have been used as feeders to support long-term survival of human epithelial cells or tissue progenitor cells (20, 21, 39). Together with our results, these facts support the idea that factor(s) secreted from primary or immortalized MEFs can be cross-reactive to human cells.

## Discussion

Features of infused cells such as sufficient number of antigen specific T cells, capabilities of proliferation and long-term persistence, successful tumor infiltration, and overcoming the immunosuppressive tumor microenvironment are associated with clinical benefit from ACT. In the current study, we provided a novel method to generate potent CD8^+^ T cell clones using NIH3T3-CM. NIH3T3-CM augmented effector functions of CTLs during initial priming and conferred memory associated-characteristics to direct long-lived memory cell differentiation. Furthermore, NIH3T3-CM programmed CTLs to reduce PD-1 expression in response to tumor antigens. Consequently, adoptive transfer of NIH3T3-CM-programmed CTLs exerted great therapeutic effects on solid tumors. Although NIH3T3 cells are murine derived cells, NIH3T3-CM could promote the differentiation of human CD8^+^ T cells into central memory and/or memory stem T like cells during antigen priming. NIH3T3-CM modulated CTLs with functionality superior to medium alone-cultured CTLs. Therefore, NIH3T3-CM provides a new insight to *ex-vivo* culture system to generate potent T cell clones for ACT.

In this study, we found that NIH3T3-CM augmented antigen induced acquisition of potent effector CTL clones by characterizing levels of effector molecules production, phenotypic expression, transcriptional regulation, and cytolytic function. Although many studies have reported that fibroblasts and fibroblast like mesenchymal stem cells play immunosuppressive roles on T lymphocytes (40, 41), our findings support the idea that embryonic fibroblasts also have the ability to strengthen effector function of T cells (19). Our results are partially in accordance with some groups suggesting that conditioned medium from lung tumor associated fibroblasts or normal (skin or lung) fibroblasts can enhance IFN-γ production of CD8^+^ T cells (16).

During initial TCR stimulation, we found that NIH3T3-CM provided signals that could enhance effector function of CTLs and modify intrinsic properties of CTLs to develop into memory-fated precursors. We further demonstrated that NIH3T3-CM-programmed CTLs, compared to medium alone-cultured CTLs, had higher capacity in long-term persistence and conversion into central memory cells upon transfer into naïve mice (Figure 3). Although phenotypic analysis showed that both medium alone and NIH3T3-CM-cultured CTLs displaying surface markers (CD127^+/−^CD27^++^KLRG1^−^) were partially associated with MPECs (CD127^++^CD27^++^KLRG1^−^) (Figure 2), NIH3T3-CM-cultured CTLs expressed higher levels of central memory markers (CD62L, CD44, and CD122). This supports the idea that *ex-vivo* expanded T cells acquiring phenotypic properties of central memory cells have high potential of memory pool formation (8, 42, 43). Furthermore, expression of CD62L on effector CD8^+^ T cells plays an important role in facilitating cells entry into secondary lymphoid or inflamed tissues (44). Consistent with this notion, high levels of CD62L may promote NIH3T3-CM-cultured CTLs homing to peripheral lymphoid organs where they reside to further develop to long-lived central memory cells under antigen free condition or they are rapidly stimulated after antigen re-encounter. Transcriptional programs that control fates of effector and memory CD8^+^ T cell were also observed. Consistence with the notion that Eomes are preferentially increased relative to T-bet in memory cell development (12, 28, 45), we observed that NIH3T3-CM dramatically enhanced levels of Eomes whereas medium alone-cultured CTLs always kept very low levels of Eomes during TCR stimulation (Figure 2C). In addition, NIH3T3-CM elevated Bcl-6 levels (Figure 2D). These results indicate that NIH3T3-CM-induced transcriptional programming plays a crucial role in the development of memory-fated clones.

We also validated that murine derived NIH3T3-CM could direct differentiation of human CD8^+^ T cells into memory-phenotype cells which showed increased expression of CCR7 and CD45RO. Primary mouse embryonic fibroblasts or their immortalized cell lines including 3T3 cells are widely utilized as feeders to facilitate self-renewal of human primary cells (20, 21, 38, 39). Our findings are consistent with previous studies because self-renew is a significant character of memory cells.

Although adoptive cells after transfusion tenaciously have survived and infiltrated to tumors, another obstacle to successful cancer immunotherapy is overcoming the immunosuppressive environment of the tumor. Exhaustion markers PD-1 and Tim3 are highly expressed on tumor infiltrating cells, leading to promotion of immune evasion of tumor cells (46, 47). In this study, we found that NIH3T3-CM-educated CTLs effectively regressed tumor growth due to their ability of survival and their prominent tumor reactivity. In tumors, we also observed that NIH3T3-CM-educated CTLs expressed low levels of PD-1 whereas almost all medium alone-cultured CTLs expressed high levels of PD-1. The same pattern was also observed on transfused cells that migrated to the spleen (Figures 5C,D). These results indicate the superior antitumor immunity of NIH3T3-CM-educated CTLs is also contributed by their features of low expression of PD-1. Another significant point of our study is that NIH3T3-CM could directly suppress PD-1 expression. It has been reported that Blimp-1 can repress CTLs expression of PD-1 during acute viral infection (48). Therefore, NIH3T3-CM-mediated suppression of PD-1 expression might be influenced by up-regulation of Blimp-1. Consistence with the notion that PD-1 is transiently expressed on CTLs after early activation and then down-regulated after antigen clearance (37), we observed that PD-1 was rapidly decreased on NIH3T3-CM-educated CTLs following transfer to antigen-free mice while medium alone-cultured CTLs showed slow PD-1 down-regulation. Moreover, NIH3T3-CM-educated CTLs kept low levels of PD-1 whereas medium alone-cultured CTLs expressed high levels of PD-1, although they were in the same tumor microenvironment (Figures 6C,D). These results imply that NIH3T3-CM-programmed CTLs with characters of memory cells might have potential of resistance of PD-1 expression whereas medium cultured CTLs with low ability of memory cell differentiation are inclined to express high level of PD-1 in tumor microenvironment. In addition, a recent report has shown that CD8^+^ TILs fail to infiltrate to tumor islands due to PD-1/PD-L and FAS/FAS-L induced apoptosis by tumor associated fibroblasts (TAFs) (49). This indicates that NIH3T3-CM-programmed CTLs with the intrinsic characteristic of low PD-1 expression may escape TAF induced apoptosis.

Although we have not yet identified the effective factor(s) derived from NIH3T3, our current results on NIH3T3-CM together with our previous investigations of MEF-CM (19) suggest that the factor(s) (not cytokines) involved in the modulation of CTL differentiation may not be a single factor. MEF-CM and NIH3T3-CM and some adult fibroblast-CM (16) may share similar factor(s) that enhance CTL effector functions. Especially, factor(s) derived from MEFs might be mainly responsible for enhancement effector function of CTLs while unique factor(s) derived from NIH3T3 might direct memory programming of CTLs. Future studies focusing on growth factors, extracellular matrix proteins, and exosomes are needed to identify soluble molecules.

In conclusion, NIH3T3-CM-educated CTLs exhibited characteristics related to memory lineages which enabled their differentiation to long lived-central memory cells after adoptive transfer. NIH3T3-CM also could program CTLs to reduce expression of PD-1 in response to tumor. Three different therapeutic models powerfully demonstrated that NIH3T3-CM-educated CTLs could efficiently regress tumor growth with high potential for ACT even if the number of transferrable cells was limited. Although soluble factor(s) underlying modulation CD8^+^ T cells remains to be identified, our findings provide a promising strategy to establish highly efficient CTL clones for ACT.

## Ethics Statement

The experimental protocols adopted in this study were approved by the Institutional Animal Care and Use Committee of Korea University

## Author Contributions

YQ and S-HP: conception and design, development of methodology, analysis and interpretation of data, and writing, review, and/or revision of the manuscript. YQ, YL, and JS: acquisition of data. JS, JHS, and TK: administrative, technical, or material support. S-HP: study supervision.

### Conflict of Interest Statement

The authors declare that the research was conducted in the absence of any commercial or financial relationships that could be construed as a potential conflict of interest.
