# Supplementary material for: NIH3T3 Directs Memory-Fated CTL Programming and Represses High Expression of PD-1 on Antitumor CTLs
Source: Front Immunol. 2019 Apr 11;10:761. doi: 10.3389/fimmu.2019.00761 (PMC6470252; doi:10.3389/fimmu.2019.00761)
Supplement: Supplementary file 1 [file Data_Sheet_1.docx]

Supplementary Material

Article Title

Yingyu Qin *, Yuna Lee, Jae Ho Seo, Taehyun Kim, Jung Hoon Shin

*** Correspondence:** Se-Ho Park, [sehopark@korea.ac.kr](mailto:sehopark@korea.ac.kr).


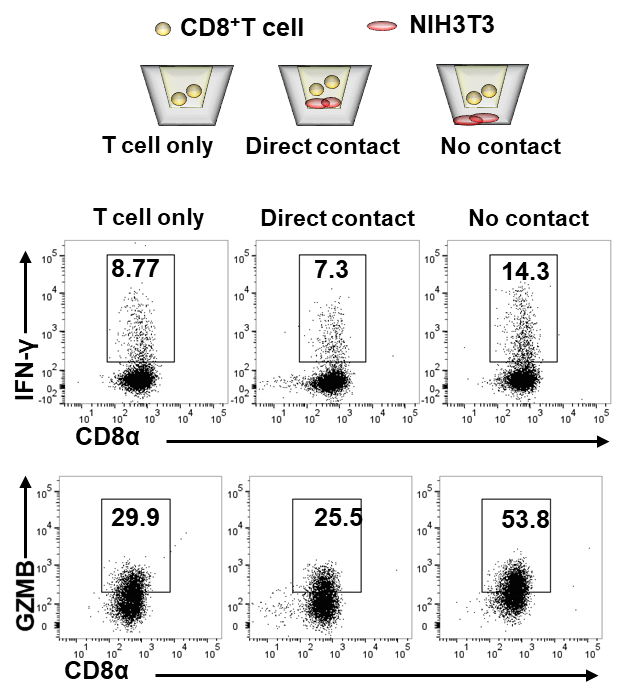


**Supplemental figure 1**. OT-1 CD8^+^ T cells (1×10^5^) in upper chamber only (T cell only) or were co-cultured with NIH3T3 cells (1×10^5^) also in upper chamber performing direct contaction (Direct contact) or separatively co-cultured in lower chamber (No contact). IFN-γ and gramzyme B (GZMB) expression levels in CTLs were detected for 2 days stimulation followed by intracellular staining.


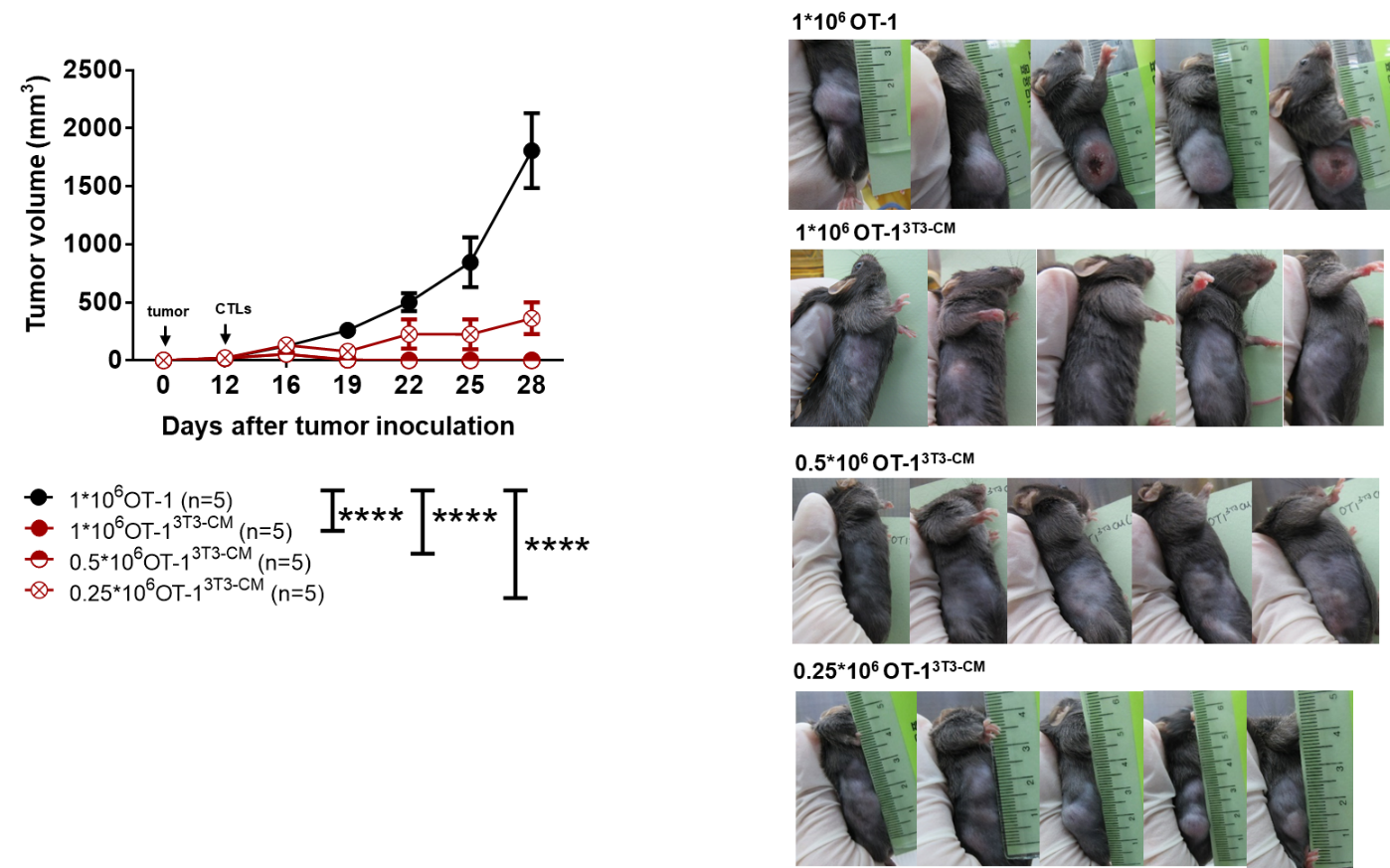


**Supplemental figure 2**. EG.7 tumor cells were subcutaneously transferred to WT B6 mice, after 30-50mm^3^ size of tumor was established, serial dilution of NIH3T3-CM cultured OT-1 CTLs (1×10^6^-0.25×10^6^) and medium alone cultured OT-1 CTLs (1×10^6^) were intravenously transferred to tumor bearing mice respectively. Then tumor growth was detected. Tumor photos were obtained after 22 days of tumor cell transfer. *****p* <0 .0001.
